# Supplementary figures and images for: Visualization of Trypanosoma brucei flagellar pocket collar biogenesis identifies two new cytoskeletal structures
Source: PLoS Biol. 2025 Oct 9;23(10):e3003429. doi: 10.1371/journal.pbio.3003429 (PMC12527162; doi:10.1371/journal.pbio.3003429)

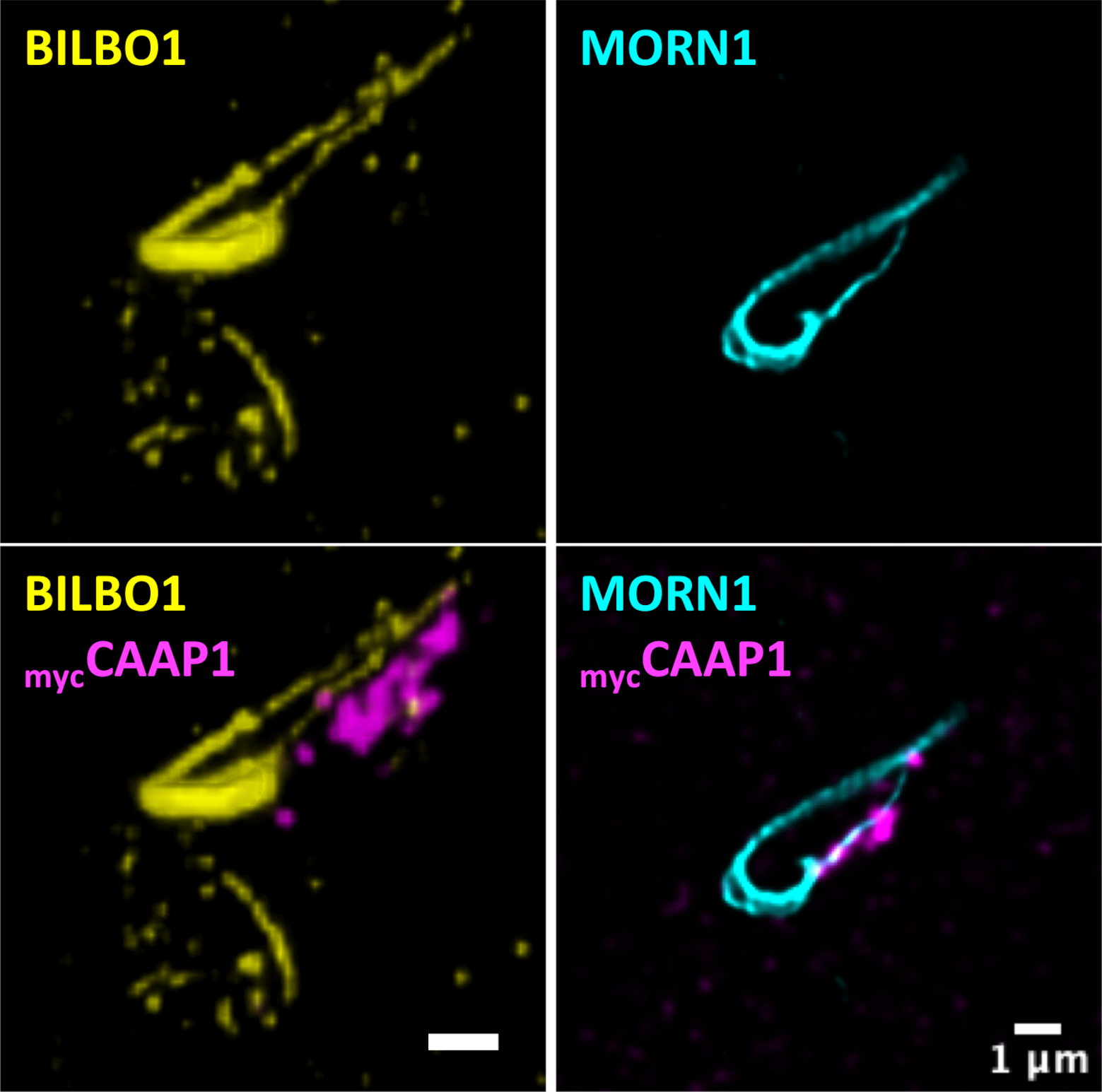

Supplement: S1 Fig — Scale bars: 1 μm. (TIF) [file pbio.3003429.s001.tif]

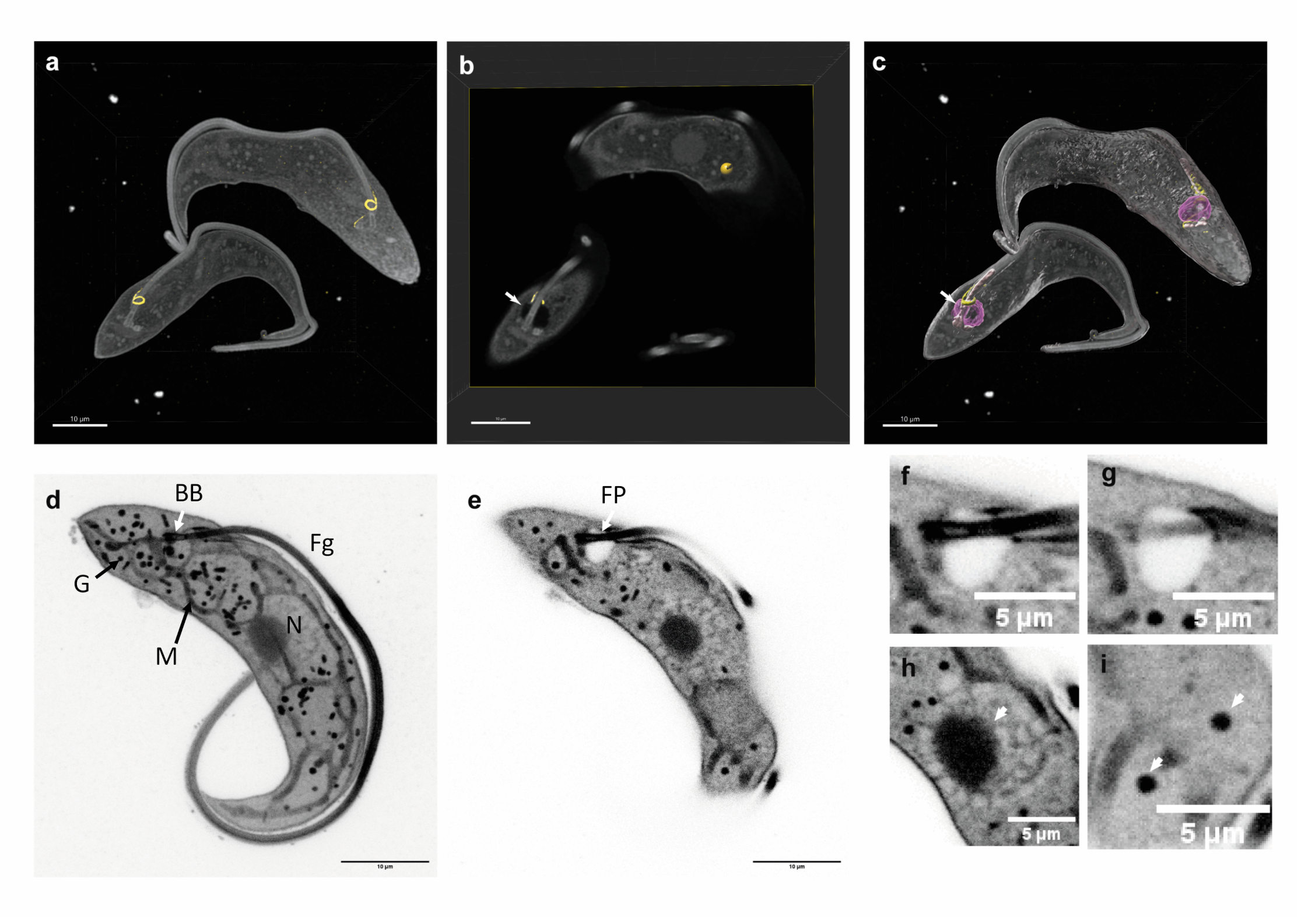

Supplement: S2 Fig — a 3D rendering of two cells labelled with NHS ester (gray) and an antibody against BILBO1 (yellow). b A single Z-plane of the same data viewed through the orthoslicer. The arrow points to the flagellar pocket (FP), which is not visible in whole-cell rendering. c Segmentation of the data shown in (a) and (b). The cell surface (transparent gray) was segmented from the NHS ester signal by intensity thresholding. Microtubule-based structures at the flagellum base (solid gray) were segmented by manual masking combined with intensity thresholding. The FP (purple, arrow) was modelled by manual segmentation, and the BILBO1 signal (yellow) was segmented by intensity thresholding. d Maximum intensity projection of NHS ester labelling (inverted LUT). NHS ester labels organelles and structures within the cell, such as the nucleus (N), mitochondrion (M), glycosomes (G), the flagellum (Fg), and the basal bodies (BB). e A single Z-plane of the data in (d). Slicing through the volume reveals additional details, such as the shape of the flagellar pocket (FP). f-i Details of single Z-planes of the data represented in (d) and (e). f,g Details of the flagellar pocket. h Nucleus with visible nucleolus (arrow). i Detail of glycosomes (arrows). Scale bars: 10 μm in a-e, 5 μm in f-i. (TIF) [file pbio.3003429.s002.tif]

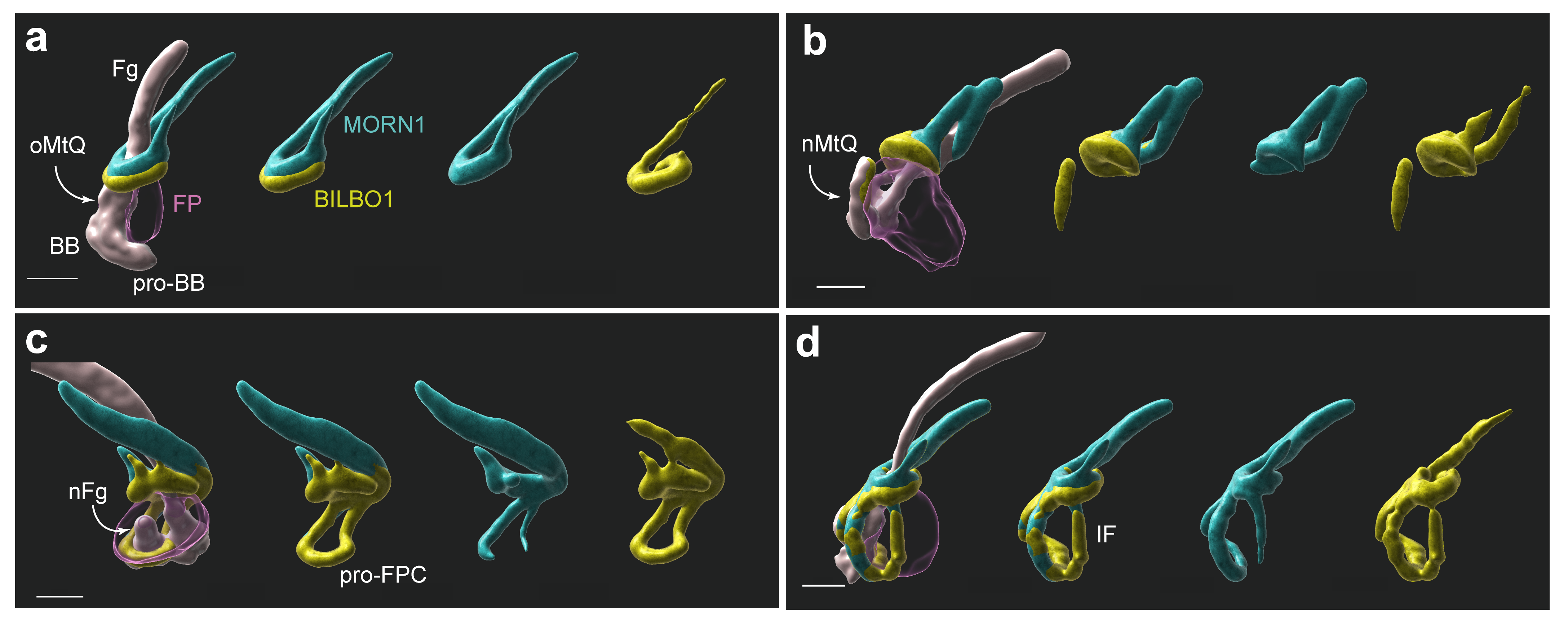

Supplement: S3 Fig — Here, BILBO1 (yellow) is tagged with a C-terminal 3xHA-tag and labelled with an anti-HA antibody. MORN1 (cyan) is labelled with a MORN1 antibody. Scale bar: 2 µm physical distance (correspond to 0.43 µm after correction for the 4.6 fold expansion factor). a—Stage 1. b—Stage 2. c—Stage 4 during new flagellar pocket collar (pro-FPC) formation. d—Stage 4 with the interconnecting fibre (IF). (TIF) [file pbio.3003429.s003.tif]
