# Supplementary material for: Visualization of Trypanosoma brucei flagellar pocket collar biogenesis identifies two new cytoskeletal structures
Source: PLoS Biol. 2025 Oct 9;23(10):e3003429. doi: 10.1371/journal.pbio.3003429 (PMC12527162; doi:10.1371/journal.pbio.3003429)
Supplement: S1 Table — This table provides detailed information on all primary and secondary antibodies, as well as NHS ester-based fluorescent reagents, used throughout the experiments. For each item, the table lists the target or label, host species (for antibodies), fluorophore, supplier, catalog number or reference, and dilution or concentration used. (PDF) [file pbio.3003429.s004.pdf]

Table S1. Antibodies used in non-expanded and expanded samples.

Antibody	Provider / Reference	Dilution Non-expanded	Dilution U-ExM	Figure
**Primary antibodies**				
MORN1 rabbit polyclonal	[1]	1:4,000	1:2,000	All MORN1 figures
BILBO1 rabbit polyclonal (anti aa 1-110)	[2]	1:2,000	1:1,000	All BILBO1 figures except Figure 3
BILBO1 rabbit polyclonal	[3]		1:500	Figure 3, Figure S3
Rabbit anti-HA	GeneTex GTX115044		1:500	Figure 1, Figure 6
Mouse anti-HA	HA-7, Sigma H9658		1:5,000	Figure S3
Mouse anti-tubulin	DM1A, Sigma T9026		1:100	Figure 1, Figure 6
Mouse anti-myc	Ozyme 2276S		1:500	Figures 6, 7
Mouse anti-myc 9E10	DSHB		1:250	Figure S1
Mouse anti-HA	Santa cruz 7392		1:200	Figure 7
Guinea pig anti-tubulin	AA345-GP Geneva Antibody Facility		1:500	Figures 2, 6
**Secondary antibodies**				
anti-mouse A488	Molecular Probes A11001		1:200	Figure 1, Figure 6
anti-mouse A647	ThermoFischer A21235		1:500	Figures 6, 7, S3
anti-mouse A546	ThermoFischer A11030		1:500	Figure 7
anti-rabbit A594	ThermoFischer A11012		1:500	Figure 1, Figure 6
Anti-rabbit A555	ThermoFischer A21428		1:500	Figure S3
anti-rabbit A488	ThermoFischer A11008		1:500	Figures 2, 4, 6, 7, S2
anti-rabbit A647	ThermoFischer A21244		1:500	Figure 2
anti-guinea pig A488	ThermoFischer A11073		1:500	Figure 2
anti-guinea pig A647	ThermoFischer A21450		1:500	Figures 2, 4, 6
**NHS Ester**				
ATTO 594 NHS ester	Sigma 08741		20 µg/mL in PBS	Figures 3, 4, S2
ATTO 488 NHS ester	Sigma 41698		20 µg/mL in PBS	Figure S3

References :

1. Morriswood B, Havlicek K, Demmel L, Yavuz S, Sealey-Cardona M, Vidilaseris K, et al. Novel Bilobe Components in Trypanosoma brucei Identified Using Proximity-Dependent Biotinylation. Eukaryot Cell. 2013;12: 356–367. doi:10.1128/EC.00326-12

2. Florimond C, Sahin A, Vidilaseris K, Dong G, Landrein N, Dacheux D, et al. BILBO1 Is a Scaffold Protein of the Flagellar Pocket Collar in the Pathogen Trypanosoma brucei. PLoS Pathog. 2015;11. doi:10.1371/journal.ppat.1004654

3. Esson HJ, Morriswood B, Yavuz S, Vidilaseris K, Dong G, Warren G. Morphology of the Trypanosome Bilobe, a Novel Cytoskeletal Structure. Eukaryotic Cell. 2012;11: 761–772. doi:10.1128/EC.05287-11
