# Supplementary material for: Visualization of Trypanosoma brucei flagellar pocket collar biogenesis identifies two new cytoskeletal structures
Source: PLoS Biol. 2025 Oct 9;23(10):e3003429. doi: 10.1371/journal.pbio.3003429 (PMC12527162; doi:10.1371/journal.pbio.3003429)
Supplement: Attachment — Submitted filename: Response to reviewers PBIOLOGY-D-25-01442R1 .docx [file pbio.3003429.s008.docx]

Response to reviewers PBIOLOGY-D-25-01442R1

**Flagellar pocket collar biogenesis: Cytoskeletal organization and novel structures in a unicellular pathogen**

Zelená Marie, Casas Elina, Lambert Chloé, Landrein Nicolas, Dacheux Denis, Bertiaux Eloïse, Abesamis Kim I, Dong Gang, Varga Vladimir, Robinson Derrick R, Bonhivers Mélanie

5th of September 2025

REVIEWS:


Reviewer #1: The trypanosome flagellar pocket with its pocket collar is a unique structure of Kinetoplastids, whose function, assembly and composition is still not fully understood. In this publication, the authors have analysed the division of the trypanosome flagellar pocket with all the attached features (hook complex, flagellar pocket collar, basal bodies, flagella). They employed UExM on detergent extracted cytoskeleton and whole cells, in combination with NHS total protein stain and a range of specific antibodies. The revisited the BILBO1 RNAi phenotype and could describe the function of BILBO1 more accurately.
This work constitutes an impressive bit of excellently-performed cell biology on a highly complex 3D structure. The authors describe the division in novel detail, and discover a few new sub-structures, such as the flagellar-pocket-collar-interconnecting fibre (a transient structure present during division). They show that the FPC remains tubulin-negative during the entire cell cycle. This work is an excellent addition to the trypanosome cell biology, and I have only a few minor comments.

*	Introduction: could the authors state on how unique the flagellar pocket collar is, given that flagellar pockets appear to be present in many cells?

We thank the reviewer for this constructive comment.

We have included (line 106):

“*While flagellar or ciliary pockets are present in many eukaryotic cells (7), including mammalian primary and motile ciliated cells where they support cilia-associated vesicular trafficking (8), the FPC is a unique cytoskeletal structure exclusive to kinetoplastids. It forms a stable, electron-dense ring that encircles the distal end of the FP and plays an essential role in FP biogenesis, membrane trafficking, cell viability and pathogenicity (4,9)*. "

*	Figure 6: The measurements appear to be based on 2-3 single measurements, it would benefit from a larger number of measurements and a bit of statistics.

We thank the reviewer for this constructive feedback. We have now increased the number of measurements for each condition in Figure 6 and included a graph summarizing the data in the revised panel 6E. Statistical details are now provided in both the figure legend 6E and described in detail in the Materials and Methods section. These additions improve the quantitative support for our conclusions.

*	MORN1 and BILBO1 give rather similar stains, indicating (partial) colocalization, but the authors could not directly show this, due to technical issues. I do not know the nature of the technical issues, but if these are minor and could be solved (for example by creating a new cell line with a different epitope tag etc), it would be nice to see the direct comparison between these two proteins.

We thank the reviewer for their constructive suggestion regarding the co-localization of BILBO1 and MORN1 through double labelling. Unfortunately, technical limitations prevented us from initially performing this experiment. High-quality antisera for both proteins could only be generated in rabbits, making double labelling unfeasible due to species-species cross-reactivity.

Moreover, previous work from Sajko et al 2020 (DOI 10.1371/journal.pone.0242677) showed that tagging MORN1 can impair its function, underscoring its sensitivity to epitope modification. For this reason, we considered MORN1 endogenous tagging or ectopic expression unsuitable. We also previously generated two BILBO1 endogenously tagged cell lines (HA and myc-tagged), but both exhibited non-negligible phenotypic alterations, likely due to interference with BILBO1 function. Given the central role of BILBO1 in FPC architecture, we chose not to pursue further analysis with these cell lines to avoid artefactual interpretation.

However, having mentioned these points, during the period of manuscript revision, we proceeded with U-ExM and co-labelling experiments on the BILBO1 HA-tagged whole cell expanded samples. These new data, now presented in Figure S3 and described in lines 310-317, demonstrate that MORN1 consistently associates with BILBO1-positive structures throughout the cell cycle: “*This hypothesis was supported by observations in a cell line expressing HA-tagged BILBO1 (Figure S3). Co-labelling experiments, combined with NHS-ester staining, revealed that MORN1 consistently colocalizes with BILBO1-positive structures throughout the cell cycle. However, we detected notable phenotypic alterations in this cell line, indicating that the tag may interfere with BILBO1 function. Given the central role of BILBO1 in FPC architecture, we chose not to pursue further analysis with this cell line to avoid misinterpretation due to potential tagging artefacts.*”

*	Line 186: "With the exception of the G1-phase cells (where no nFPC is being formed) and the period when the FPC-IF is detected, the oFPC consistently accounts for approximately 40% of the total BILBO1 fluorescence, remaining close to 50% throughout the cell cycle. This indirectly suggests that nFPC biogenesis occurs de novo, rather than through redistribution of BILBO1 from the oFPC." I failed to understand this: If there would be redistribution of BILBO1 from the oFPC rather than de novo assembly, why would you expect different percentages?

We thank the reviewer for highlighting the need for clarification. Our reasoning is based on the hypothetical outcome if BILBO1 were redistributed from the old FPC to the forming nFPC, without any new BILBO1 incorporated into the oFPC, and in a semi-conservative way. In such a scenario, we might expect that at some point the BILBO1 signal at the oFPC to decrease during nFPC formation. However, this was not clearly conveyed in the original text.

To address this, 1) we have added the quantitative analysis (mean ± standard deviation) and number of cells measured in the legend of figure 2G and 2) revised the text for clarity, as follows (lines 241-246): “*Quantification of BILBO1 labelling at the oFPC and at the area of nFPC formation (MtQ, proFPC, FPC-IF) reveals that an equivalent amount of BILBO1 is present in the newly forming structures compared to the mature oFPC (Figure 2G). Given that the early stages of nFPC biogenesis occur at the level of the basal body, and that no reduction in BILBO1 is observed at the oFPC, these findings suggest that nFPC formation involves de novo recruitment of BILBO1, rather than redistribution from the existing structure.*”

We hope this revised explanation and the accompanying figure improve clarity and address the reviewer’s concern.

*	Given that this is a rather complex, 3-dimensional structure: would it be possible, to include a model of the pocket division to the discussion part, summarising all the major structures and findings? This would help the non-expert readers a lot.

We thank the reviewer for this thoughtful suggestion. We agree that a schematic model summarizing the 3D organization of the flagellar pocket and associated structures could aid non-expert readers. Figure 8 was designed with this intent in mind and provides a simplified 2D representation to highlight the sequence of the structural events during FPC biogenesis. However, we acknowledge that this figure does not fully capture the three-dimensional intricacies of the system, particularly those of the FP itself. Creating a comprehensive 3D model that accurately conveys these features would require considerable time and specialized illustration expertise to ensure both clarity and accuracy. Unfortunately, such resources are beyond our current capacity. Nevertheless, and, following reviewer's 3 comment on Figure 8, we hope that the expanded legend of Figure 8 will better serve as a useful framework for readers and provide a foundation for future refinement as our understanding of these structures deepens.

*	Line 126 (130 on my word version): 	Typo (expressing)

corrected


**Reviewer #2:** This paper describes a microscopy-based analysis of biogenesis of one of the key vital cytoskeletal structures in Trypanosoma brucei parasites, the hook complex/flagellar collar structure.

The paper is very single method-focused, but applies it very well, making use of the power of expansion microscopy. This is a powerful application, as these are highly structured structures which are on the edge of being resolved by conventional light microscopy, but well-resolved by expansion microscopy, maximising the value of this approach. Also, some of these structures, particularly the hook complex, are not readily visible by electron microscopy, making this a powerful approach for targeted consideration of known proteins. This comprehensively maps how this vital structure duplicates for cell division, providing insights into complex flagellar pocket morphogenesis. 

A limitation is that expansion microscopy cannot distinguish between assembled material and material which is in transient structures or simply being transported. Flux of material will bias the visualised protein to assembled material, but this is a limitation. It was not always clear whether samples for particular experiments are cytoskeletons or whole cells, where material in transport is more likely to be extracted. Please try consistently indicate in the text and figures.

We thank the reviewer for raising this important point regarding the limitation of expansion microscopy in distinguishing between assembled and transient or transported material. We agree that this distinction is essential for interpreting protein localisation, and we recognize that expansion microscopy is biased towards detecting assembled or more stable structures.

To improve clarity, we have consistently indicated whether observations were made in whole cells or cytoskeletons throughout the manuscript. We have reviewed the text and figure legends to ensure that this distinction is now clearly and consistently noted throughout the manuscript and figures.

It is powerful that BILBO and MORN are antibody to native protein, avoids problems with assembly differences for tagged/untagged allele, or subtle defects due to a tag. However, more care should be taken describing the microtubule quartet, as it extends along the entire FAZ. The SPEF1 positive portion (and BILBO/MORN association) is only the proximal microtubule quartet, and that should be made clear whenever the microtubule quartet is discussed (given the abbreviation-heavy text, pMtQ for proximal microtubule quartet might be useful).

We thank the reviewer for this helpful observation. To avoid confusion, we have revised the manuscript to explicitly state that our observations pertain to the proximal portion of the MTQ wherever relevant. Given the already abbreviation-heavy nature of the text, we have chosen not to introduce an additional abbreviation, but have instead clarified the context directly in the text:

Lines (54,112,134,160,177, 210, 330).

Much as the core data is qualitative. However, all figures would benefit from sample size indications. Making it clear from how many examples the selected example is representative of - both number of sample preparations and number of cells from which the example was selected.

We thank the reviewer for this important suggestion. Despite the increase in resolution provided by ExM with the related restricted ability to image large fields of view we were able to image tens of cells at each stage, in total over 200 cells, making this approach quantitative. We have now included specific sample size for information in the figure legends.

For example:
Figure 1: For each, representative example from how many. But, this matches tomography data, so less important to have high confidence, it meets expectations.

We thank the reviewer for this important suggestion. The data were collected from 5 independent expanded gel preparations yielding a total of 53 images distributed across cell cycle stages (4 images representative of a, 27 representative images of b-e, 22 representative images of g). This information has been added to the legend of figure 1.

Figure 2: For each, representative example from how many. Particularly important to give confidence in the complex structures in stages 4, 5.

The data were collected from 10 independent expanded gel preparations yielding a total of 193 images distributed across cell cycle stages as follows: 9 images for stage 1, 47 for stage 2, 38 for stage 3, 38 for stage 4, 40 for stage 5, 21 for stage 6. This information has been added to the legend of figure 2.

Figure 3: A, representative example from how many, B, sample size per data point.

The data were collected from 7 independent expanded gel preparations yielding a total of 48 images distributed across cell cycle stages as follows: 7 images for stage 1, 5 for stage 2, 8 for stage 3, 5 for stage 4, 11 for stage 5, 12 for stage 6. This information has been added to the legend of figure 3. We have increased the number of measured cells in which the parameters were measured to 5 for each stage.

Figure 4: For each, representative example from how many. Like Figure 3, confidence in the stage 4 and 5 examples.

For figure 4A: The data were collected from 4 independent expanded gel preparations yielding a total of 69 images distributed across cell cycle stages as follows: 9 images for stage 1, 8 for stage 2, 12 for stage 3, 15 for stage 4, 12 for stage 5, 13 for stage 6.

For figure 4B: The data were collected from 3 independent expanded gel preparations yielding a total of 22 images distributed across cell cycle stages as follows: 5 images for stage 1, 3 for stage 2, 4 for stage 3, 2 for stage 4, 1 for stage 5, 7 for stage 6.

This information has been added to the legend of figure 4.

We hope that this clarification provides the necessary context to evaluate the reproducibility and representativeness of the presented data.

Figure 6: Represantative of how many, and preferably add a mini bar chart of separation with a few data points per measurement.

We thank the reviewer for this constructive feedback. We have now increased the number of measurements for each condition in Figure 6 and included a graph summarizing the data in the revised panel 6E. Statistical details are now provided in both the figure legend 6E and described in detail in the Materials and Methods section (line 791). These additions improve the quantitative support for our conclusions.

Overall I found the text well written, easy to understand, well cited, etc. including the hard to describe complex three dimensional rearrangement in the results. I have a few minor comments about phrasing descriptions in the introduction and discussion.

Introduction
Line 75: Not well supported to describe the cortical cytoskeleton as a limitation. May well be a key advantage in different areas, and advantageous to concentrate all endo/exocytosis in one structure.

We thank the reviewer for this insightful comment and have clarified this point in the revised manuscript lines 92-102:

“*Trypanosome morphology and biochemistry have revealed a dense subpellicular microtubule cytoskeleton that forms a corset-like structure, restricting vesicular trafficking across most of the cell surface (1). This tightly cross-linked cortical cytoskeleton precludes processes such as endocytosis, exocytosis, and phagocytosis at sites other than the flagellar pocket (FP), a specialized invagination of the plasma membrane at the base of the flagellum. As such, the FP serves as the sole site for endo- and exocytosis and is essential for nutrient uptake, protein secretion, immune evasion, and cell morphogenesis (2–4). This architectural constraint likely reflects an evolutionary trade-off: limiting dynamic membrane trafficking elsewhere in order to maintain a highly organized and efficient system centred on the FP. This specialization enables critical adaptations for parasite survival, including hydrodynamic flow-based clearance of immune complexes and rapid turnover of surface proteins (2,3)*”.

Line 81: May be worth noting similarity/analogy to feeding grooves and similar structures at the base of flagella in free living flagellates. Not vital though.

We thank the reviewer for this insightful suggestion. Indeed, the analogy between the FP and feeding grooves or other flagellum-associated invaginations observed in free-living flagellates is an interesting point of evolutionary and functional comparison. While this is beyond the current scope of our manuscript, we agree that it highlights the broader relevance of flagellum-associated structures in eukaryotic cell organization and may serve as a useful perspective for future comparative discussions.

Line 86: Formally, the microtubule quartet is conserved, but there is diversity around this. The cytostome microtubules in cruzi and cytostome-containing monoxenous species like Paratrypanosoma, the additional pocket and cytoplasmic microtubules in Leishmania. More nuance would be good.

We thank the reviewer for this important clarification. We agree that while the microtubule quartet is a conserved cytoskeletal element in kinetoplastids, its morphological context and functional associations differ across species. In particular, we acknowledge the variations observed in *T. cruzi*, *Leishmania*, and monoxenous species such as *Paratrypanosoma*, where related microtubule structures are involved in cytostome architecture or associate with multiple flagellar pockets. We have now revised the relevant sentence in the Introduction (lines 113-132) to better reflect this diversity:

" *Originally described in T. brucei (5), the MtQ is a conserved feature among kinetoplastids but its organization and associations can differ substantially. For example, in T. cruzi and Paratrypanosoma, MtQ-related microtubules contribute to the cytostome architecture, and Leishmania species exhibit additional cytoplasmic and pocket-associated microtubules (11–14).*"

We hope this added nuance appropriately addresses the reviewer’s concern and improves the manuscript.

Line 88: Again, clarify about whether this is formally known for all trypanosomatid species, as the text structure implies that it is true/known.

Where relevant, we have adjusted the text to clarify the species studied and avoid overgeneralization.

Line 105: Clarify species where this evidence comes from, *ie. T. brucei.*

We have clarified this by writing (line 153):

*“Tomography studies on T. brucei have characterized...”.*

Line 110: State "in *T. brucei".*

We have clarified this by writing (now line 158):

*“In this study, we characterize the T. brucei the cell cycle-related...”.*

Line 342: An extremely early event is Aurora Kinase association with the MtQ, worth quickly incorporating this point.

We thank the reviewer for this suggestion. Several kinases have been identified to target the MtQ; for example, TbPLK and TOEFAZ have been identified on the FAZ/MtQ, with TbPLK migrating to the FPC, HC, FC, and the growing tip of the new FAZ. Aurora kinase homologs and components of the chromosomal passenger complex have been shown to migrate from the nucleus to the new FAZ tip. However, we did not find data on Aurora Kinase association with the MtQ. We are unsure, but perhaps, the reviewer may be referring to TbPLK, as we are not aware of published evidence for Aurora kinase association with the MtQ in *T. brucei*. TbPLK has indeed been shown to associate early with the MtQ and plays a role in flagellar pocket biogenesis. We have now incorporated this point into the Introduction to highlight the early involvement in MtQ-associated events (see line 137): *“Importantly, in T. brucei, a Polo-like kinase (TbPLK) is one of the earliest proteins to associate with the new forming MtQ during the cell cycle (15)*.”

If the reviewer was instead referring to a different study or specific Aurora kinase data, we would be grateful for clarification or guidance on the reference.

Line 353: Some more clarity about force generation here would be useful. The procyclic form flagella connector, vs. bloodstream form groove may provide a force.

We thank the reviewer for raising this important point. While our study focused on structural dynamics during FPC and FP formation, we agree that the mechanisms of force generation underlying basal body and FPC movement warrant further discussion. In the revised manuscript, we now mention that in procyclic forms, the flagella connector has been proposed to generate or transmit forces that guide basal body positioning and flagellum elongation (Moreira-Leite et al., 2001). However, bloodstream forms lack a flagella connector, and instead rely on a membrane groove (Smithson et al, 2022), which may provide an alternative structural interface or mechanical constraint.

We have incorporated this expanded discussion into the revised manuscript (Lines 499–531) to reflect these considerations and acknowledge the potential structural differences between life cycle stages in force generation mechanisms (line 531-593): “*It is unclear if the proFPC is involved in ridge formation in the early stages of pocket division. However, we propose that BILBO1, via the proFPC, is directly involved in ridge formation after new flagellum rotation, but the precise mechanisms and driving forces remain to be elucidated. However, in procyclic forms, the flagella connector (FC), an electron-dense structure linking the tip of the new flagellum to the lateral aspect of the old flagellum, has been proposed to transmit forces that guide basal body separation and flagellar elongation (29). The timing of these events is notable: the early rotation and limited anterior displacement of the new basal body and associated structures occur while the FC is still actively migrating. This suggests the FC may contribute to positioning during early proFPC assembly. However, the more pronounced posteriorward migration of the nBB typically occurs after FC migration has ceased (39), indicating that the FC may not be the primary force-generating mechanisms throughout the entire process. In bloodstream forms, which lack a flagella connector, a distinct transmembrane structure called the groove has been described at the tip of the new flagellum (40). While this may serve as an alternative structural interface or impose mechanical constraints, its formation appears to occur only after the new flagellum has exited the FP. Therefore, its role in early BB and proFPC positioning remains uncertain. Importantly, studies in IFT20 and DHC1b RNAi procyclic cells, in which new axoneme assembly is impaired, demonstrate that BB rotation and separation can still occur in the absence of a new axoneme (39). These findings argue against a model in which the FC exerts force exclusively via the axoneme. Instead, we propose that early proFPC dynamics may rely on other mechanisms, potentially involving BB-associated motor proteins, local membrane remodelling, or cytoskeletal interactions.”*

Line 380: IFT is exceptionally specific to flagella, this would be very odd. Worth some consideration on likely trafficking direction of kinesins, although I realise this can be hard to predict.

We thank the reviewer for this valuable comment. We have modified the text to emphasize the kinesins rather than IFT:

Line 509-515:

“*While FPC and HC proteins localise to the MtQ, proFPC, and FPC-IF, the precise mechanism by which they are trafficked to these structures remains unclear. Proteins may be recruited directly from the cytoplasm or are transported along the microtubules by motors. However, several kinesins are positioned on the proximal MtQ, (35,20), and given the theoretical polarity of the MtQ, with microtubule plus-ends orientated away from basal body, their positioning is consistent with a role in anterograde transport from the basal body region towards the FPC.*”

More generally, do you think there is any informative link with potential transport of material along the entire microtubule quartet, for supporting eg. FAZ assembly or coordinating ER attachment?

We thank the reviewer for this valuable comment.

We previously noted (Line 376) “Furthermore, the absence of the flagellum attachment zone (FAZ) in BILBO1 RNAi cells (6), which leads to flagellum detachment along the cell length, suggests that the MtQ plays a key role in FAZ formation and positioning”.

While we agree that the MtQ and associated kinesins raise interesting possibilities in broader roles, such as FAZ assembly or ER coordination, these aspects fall outside the primary scope of our current study. For this reason, we have limited our discussion to the potential role of MtQ-associated kinesins in FPC and HC biogenesis. However, we have modified the text to now read (line 504-508):

“*Furthermore, it is unclear if there is a link with potential transport along the entire MtQ for supporting FAZ assembly and its ER attachment, but the absence of the FAZ and disturbed ER plus the mis-orientated MtQ in induced BILBO1 RNAi knockdown cells ((4), and this work) is in line with the potential role of the MtQ in providing a platform for transport of FAZ constituents and ER-attachment*."

Line 401: (Our model aligns with previous studies (34), showing that FP formation precedes flagellum separation during proventricular stage development. However, key questions remain regarding the forces driving ridge invagination.)

Do I understand correctly that the new flagellar pocket collar does not have a presence in the early ridge between the dividing pockets? Possibly worth reversing this statement to say that your results show that it is unlikely that collar or hook complex rearrangements are fully responsible for early ridge formation.

We thank the reviewer for this insightful comment and the opportunity to clarify our interpretation. Our statement in line 401 referred to the current understanding that flagellar pocket (FP) biogenesis is semi-conservative, and the new flagellar pocket collar (FPC) forms prior to new flagellum exit, as also supported by previous work (Lacomble et al., 2010).

Our own U-ExM data do not reveal an early ridge structure prior to stage 5. This may reflect either its actual absence at earlier stages or technical limitations, as a narrow ridge may not be readily detectable by NHS ester staining. We do, however, observe key morphological transitions between stages 4 and 5. Notably, the distance between the new and old FPCs decreases by ~1.5 µm (in expanded specimens corresponding to 1.5/4.6=0.33 µm), which we interpret as the new FPC relocating more posteriorly, potentially driven by ridge formation. Concurrently, the distance between the new basal body (nBB) and the new FPC increases by ~2.5 µm, consistent with posteriorward movement of the nBB associated with new FP emergence. These observations align well with electron microscopy data from Lacomble et al. (2010), where no ridge is seen when the new flagellum is still within the shared FP (analogous to our stage 4), and a ridge appears only after new flagellum rotation and separation (stage 5).

While BILBO1, present at the proFPC, is strongly implicated in shaping the ridge, we agree that our data suggest that neither FPC nor hook complex rearrangements alone account for its initial invagination. To improve clarity, we have revised the sentence in line 531-575 to better reflect this view:
“*It is unclear if the proFPC is involved in ridge formation in the early stages of pocket division. However, we propose that BILBO1, via the proFPC, is directly involved in ridge formation after new flagellum rotation, but the precise mechanisms and driving forces remain to be elucidated*.”

We hope this revision clarifies our interpretation of the spatial and temporal coordination of ridge formation with respect to FPC and flagellum dynamics.

**Reviewer #3** (Markus Engstler, signs review): 

General 

This is an excellent manuscript that presents a comprehensive and technically rigorous analysis of flagellar pocket collar biogenesis in Trypanosoma brucei, using advanced ultrastructure expansion microscopy.

We sincerely thank Prof Markus Engstler for his positive assessment of our manuscript. We greatly appreciate his recognition of the technical rigor and comprehensive nature of our work, and are encouraged that the study is seen as a valuable contribution to the understanding of flagellar pocket collar biogenesis.

The identification and characterization of two previously undescribed structures, the pro-flagellar pocket collar (proFPC) and the FPC-interconnecting fibre (FPC-IF), represent a major advance in our understanding of how new FPCs are assembled de novo during the cell cycle. The application of ultrastructure expansion microscopy is particularly powerful here, enabling unprecedented resolution of spatial and temporal dynamics of FPC, HC, and MtQ components. These discoveries revise and extend existing models of FPC biogenesis and may also provide broader insights relevant to ciliary pocket formation in other eukaryotic systems. 

Major 

1.	Mechanistic interpretation of proFPC and FPC-IF formation
While the structural characterization of these elements is excellent, the manuscript could benefit from a more detailed discussion on potential molecular mechanisms underlying their formation. The authors briefly mention motor proteins such as TbKINX1B, but do not elaborate on possible regulatory or recruitment pathways. A more speculative discussion here would add value without requiring additional experiments.

We thank the reviewer for this thoughtful and constructive suggestion. We agree that a more speculative mechanistic discussion would enhance the impact of our findings and provide a useful framework for future studies. Some aspects of this topic also overlap with comments raised by Reviewer #2 and have been partially addressed in our response to line 380.

To address this point more comprehensively, we have now expanded the discussion to provide a speculative model integrating structural and regulatory elements underlying proFPC and FPC-IF formation (lines 653-670 of the revised manuscript):

“*We suggest that the regulatory mechanisms governing proFPC and FPC-IF formation are orchestrated by a cascade of spatially and temporally controlled protein-protein interactions. A central player in this process is BILBO1, which acts as a master organizer of FPC assembly and is recruited early to nascent sites of FPC biogenesis. Its early presence at the proFPC and nMtQ suggests it may serve as the initial structural seed for complex assembly. This may relate to BILBO1’s intrinsic ability to self-polymerize into distinct morphologies, either linear (e.g., FPC-IF) or circular (e.g., mature FPC), depending on local cues. Spatial regulation of BILBO1 polymerization may be modulated by calcium or post-translational modifications such as phosphorylation (18). The initiation of proFPC assembly likely depends on the spatial definition of nucleation sites, near the base of the forming nMtQ. Subsequent extension along the oMtQ and around the nascent FP neck may reflect guided polymerization. Additional components, such as TbKINX1B and FPC-associated proteins (20–23), may be recruited through direct interactions with BILBO1 polymers and contribute to anchoring or remodelling. Cell cycle–regulated kinases such as NRKC (involved in BB separation) (41) and TbPLK (15) (which localizes to the nMtQ early in the cycle) may further orchestrate this process by phosphorylating scaffolding or adaptor proteins that act as docking platforms. Disassembly of transient structures such as the FPC-IF may be driven by active depolymerization or destabilization events, mimicking mechanisms observed for other cytoskeletal assemblies.*”

We hope this expanded discussion offers a useful speculative framework for understanding the regulation and dynamics of FPC-related structures.

2.	Functional implications and broader context
The authors are encouraged to elaborate further on the potential biological role of the transient FPC-IF and the significance of its rapid disappearance. How might these structures contribute to flagellar pocket dynamics, immune evasion, or membrane remodeling? Even in the absence of direct experimental data, this would help position the work more broadly within parasite cell biology.

We thank the reviewer for this thoughtful suggestion to elaborate on the biological implications of the FPC-interconnecting fiber (FPC-IF). We agree that contextualizing this transient structure within the broader framework of flagellar pocket (FP) biogenesis and parasite biology is important. We have accordingly expanded the Discussion to include a more speculative but integrative perspective on its potential roles.

In particular, we highlight that the FPC-IF may act as a mechanical and spatial scaffold linking the old and newly forming FPCs, thereby coordinating cytoskeletal dynamics and membrane remodeling during FP biogenesis. We also discuss how its transient nature and variable morphology may reflect functional plasticity during rapid structural transitions, such as basal body rotation. Finally, we mention that although our observations are in procyclic cells, whether this structure is conserved in bloodstream forms remains unknown, but the conservation of core FP/FPC components across kinetoplastids suggests that analogous mechanisms may exist.

Revised discussion text (lines 632-652):

“*Although direct functional evidence remains limited, the transient FPC-IF likely plays a critical role in coordinating the morphogenesis of the FPC in T. brucei. By transiently linking the old and newly forming FPCs, the FPC-IF may act as a mechanical and spatial scaffold that ensures proper positioning and segregation of key cytoskeletal components such as BILBO1 and MORN1. This linkage likely stabilizes the FP neck region during the major membrane and cytoskeletal remodelling events that accompany flagellum duplication and FP formation. The dynamic assembly of the FPC-IF may also facilitate the targeting or stabilization of membrane-associated proteins, contributing to the establishment of specialized membrane domains within the FP that are critical for its endocytic and exocytic function. As the FP serves as the exclusive site of endocytosis and exocytosis in T. brucei, including for surface glycoprotein trafficking, maintaining its integrity is essential for immune evasion. Additionally, disruption of FP biogenesis, via incorrect FPC-IF function or positioning, could compromise endocytic efficiency and immune evasion by mis-localizing surface proteins. Moreover, the precise but transient appearance of the FPC-IF at the interface of the old and new FPCs suggests a role in shaping membrane curvature and compartmentalization during FP morphogenesis. Collectively, these observations support the notion that the FPC-IF integrates cytoskeletal organization, membrane remodelling, and trafficking to ensure proper FP inheritance and functionality. The disassembly of the FPC-IF likely facilitates proper positioning of the nFPC and associated cytoskeletal components for subsequent cell division. We further speculate that analogous structures may exist in other kinetoplastids, such as T. cruzi and Leishmania, where conserved FP/FPC architecture suggests the potential for similar mechanisms*.”

3.	Clarity and terminology 
Consider standardizing terminology when referring to old/new structures (e.g., oFPC/nFPC vs. proFPC). In some instances, the transitions between stages and structures could be clearer. A table or expanded legend in Figure 8 summarizing each step in the proposed biogenesis pathway would aid readability.
We thank the reviewer for this helpful suggestion. While we recognize the importance of consistent terminology, we have opted to retain the proFPC/nFPC nomenclature, as it reflects the developmental progression from the intermediate proFPC structure, which reflects early stages, to the more mature nFPC. We believe this distinction is important for accurately conveying the sequential nature of FPC biogenesis. To further clarify these transitions, we have substantially expanded the Figure 8 legend to provide a detailed, stepwise description of each stage, including the major structures involved. We hope this revision improves both clarity and the overall readability of the proposed model.

Minor 

Line 96: "…serving as a the structural backbone…" 
We have corrected the sentence.

Abstract: Consider condensing the background portion to strengthen the focus on findings.

We thank the reviewer for this helpful suggestion. We have revised the abstract to reduce the background content and shift the emphasis more directly toward our findings, in line with the reviewer's recommendation.

A useful model figure, but the legend should be expanded to clarify each structure and its temporal context.

We thank the reviewer for this helpful suggestion. We have expanded the figure legend to clarify each structure and its temporal context.

Figure legends: Ensure all abbreviations (e.g., FC, BB, FAZ) are defined on first use in figure legends, not just the main text.

We have carefully revised all figure legends to ensure that abbreviations such as FPC, FP, HC, BB and others are now clearly defined upon their first use in each legend.

Data and methods

The methodological descriptions are thorough and appropriate. The use of detergent-extracted and whole-cell U-ExM, coupled with well-validated antibodies, is convincing. Data availability is clearly addressed.

While the morphological data and imaging results are compelling, the manuscript would benefit from greater transparency and rigor in the reporting of quantitative analyses.

Specifically:

Please clarify the number of biological replicates and individual cells measured for each quantitative analysis (e.g., fluorescence intensity in Figure 2G, distances in Figure 3B).

We have now clarified the number of biological replicates and individual cells analysed for each quantitative measurement throughout the figure legends and in the Material and Methods section.

For datasets involving continuous measurements (e.g., nBB-nFPC distances), consider reporting descriptive statistics such as mean ± SD or using graphical formats (e.g., box plots) that reflect data distribution.

We have now updated the relevant figures (figure 2G with quantification of BILBO1 labelling in the oFPC, Figure 6E with quantification of the distance between the MtQ and the BILBO1-positive structure).

Indicate whether quantification was performed in a blinded fashion and whether statistical comparisons (e.g., tests for significance between stages) were applied where appropriate.
While these additions are not critical for validating the overall conclusions, they would significantly enhance the statistical rigor and reproducibility of the study.

We have now included, in the material and methods and in the figure legends, the statistical comparisons.

Conclusion

This is a highly original and well-executed study that makes a strong contribution to the field. It is suitable for publication in PLOS Biology pending minor revisions.

We sincerely thank the reviewers for their positive and encouraging feedback. We are grateful for their constructive comments, which have helped us further improve the clarity and rigor of the manuscript.

In addition, we made minor revisions to improve the clarity and flow of the text. We also added the following sentence in the Discussion (lines 469-474) to clarify a point not fully addressed in the previous version: “*We observed a shift in the chronology of proFPC movement: in cytoskeletons it occurs from stages 3 to 4 (Figure 2), whereas in whole cells it is delayed until stages 4 to 5 (Figure 3). We propose that this discrepancy represents an artefact of detergent extraction, arising from the absence of the FP membrane, which in whole cells may normally provide slight resistance to proFPC displacement*.”
